# Estimation of Feeding Composition of Industrial Process Based on Data Reconciliation

**DOI:** 10.3390/e23040473

**Published:** 2021-04-16

**Authors:** Yusi Luan, Mengxuan Jiang, Zhenxiang Feng, Bei Sun

**Affiliations:** School of Automation, Central South University, Changsha 410083, China; 184611053@csu.edu.cn (Y.L.); jiangmengxuan@csu.edu.cn (M.J.); fengzxcsu@csu.edu.cn (Z.F.)

**Keywords:** data reconciliation, robust estimator, gross error detection, feeding composition

## Abstract

For an industrial process, the estimation of feeding composition is important for analyzing production status and making control decisions. However, random errors or even gross ones inevitably contaminate the actual measurements. Feeding composition is conventionally obtained via discrete and low-rate artificial testing. To address these problems, a feeding composition estimation approach based on data reconciliation procedure is developed. To improve the variable accuracy, a novel robust M-estimator is first proposed. Then, an iterative robust hierarchical data reconciliation and estimation strategy is applied to estimate the feeding composition. The feasibility and effectiveness of the estimation approach are verified on a fluidized bed roaster. The proposed M-estimator showed better overall performance.

## 1. Introduction

Complete, accurate and reliable data measurements are important for process modeling, model identification, real-time online optimization and process control. However, the measured values of variables in the actual measurement process are inevitably contaminated by random errors or even gross errors. Therefore, the process measurements will deviate from the real values. In addition, the feeding composition, which is important in making control decisions, cannot be measured online due to the high economic cost and technical limitations. Therefore, it is necessary to process the measurements to guarantee the reliability and observability of the system. Based on this, the estimation of feeding composition can be better performed. Data reconciliation is an effective approach for this aim [1]. The principle of data reconciliation is to minimize the sum of square deviations between the coordinated value of variables and their measurements under the condition of satisfying material balances, heat balances and boundary constraints of process variables. From the mathematical point of view, data reconciliation is a constrained optimization problem [2], which has been widely used in many important procedures such as in-line process monitoring [3,4], enhanced control strategy performance [5,6], parameter estimation [7], soft sensor applications [8], quality control [9] and industrial optimization [10,11] among others [3,4,5,6,7,8,9,10,11].

Traditional data reconciliation typically assumes that measurements contain only random errors with an average value of zero and normal distributions. Nonetheless, the measurements will be contaminated by gross errors. If gross errors are not processed, the accuracy of data reconciliation results will be reduced. The gross error detection is therefore particularly important for data reconciliation. There are currently three main methods for handling measurements with gross errors. The first strategy is sequential gross error detection and data reconciliation. A variety of statistical tests, including the global test [12], the measurement test [13], the nodal test [14], generalized likelihood ratio test [15] and principal component analysis test [16], are used for gross error detection. Following the detection and elimination of gross errors, the traditional data reconciliation is carried out. The second strategy is simultaneous gross error detection and data reconciliation. Yu et al. [17] proposed a support vector regression method for recursive simultaneous data reconciliation and gross error detection. Zhang et al. [18] used a novel particle filter (PF) algorithm based on the measurement test (MT) to solve the dynamic simultaneous gross error detection and data reconciliation. Yuan et al. [19] established a new hierarchical Bayesian framework, which can simultaneously estimate the real value of process variables and obtain the magnitudes of gross errors. The last strategy is robust data reconciliation. The estimations close to the true value can be obtained by reducing the influence of gross errors on data reconciliation results. Tjoa and Biegler [20] constructed an M-estimator based on mixed distribution, which takes both random errors and gross errors into account and minimized the M-estimator as an objective function. Johnson and Kramer [21] presented a new robust method for data reconciliation based on a probability bootstrapping theory. Arora and Biegler [22] evaluated the Fair and Hampel estimators in stationary and dynamical constrained problems. Hampel’s three-part estimator presented the best performance. Wang and Romagnoli [23] designed a partially adaptive estimator based on a generalized T distribution and a fully adaptive estimator based on density estimation by studying the robustness of a set of existing estimators. Özyurt and Pike [24] analyzed the performance of a number of robust estimators such as Hampel, Cauchy and Fair. Ragot et al. [25] adopted a cost function that has been improved based on the contaminated probability density distribution model for the design of robust estimator. Prata et al. [26] applied the proposed strategy based on the particle swarm optimization (PSO) and the robust Welsch estimator to real-time online detection of Bulk polymerization of propylene. Zhang et al. [27] constructed a robust estimator by using the quasi-weighted least squares. Through the comparative analysis of various methods like Welsch, quasi-weighted least squares and comentropy M-estimators, the feasibility and effectiveness of robust estimators in simultaneous gross error detection and data reconciliation were demonstrated [28]. Alighardashi et al. [29] proposed a maximum likelihood framework for simultaneous data reconciliation and gross error detection for steady-state data. Xie et al. [30] utilized a novel robust estimator to improve the robustness of data reconciliation. Forty-eight different robust M-estimators can be founded in a recent review study, including Fair, Cauchy, Biweight, Jin, Welsch estimator and Xie [31].

The key to estimating feeding composition based on data reconciliation is the robust estimator and the design of the estimation strategy. An excellent robust estimator is embodied in two aspects: On the one hand, the objective function is bounded, and the bound value is small; on the other hand, the influence function can converge quickly and converge to zero. The objective functions of Cauchy [32] and Fair [33] estimators increase with the increase in standard measurement residuals, indicating that the error will greatly affect the reconciled results. Besides, the convergence speed of their influence functions is very slow, and the bound values are large, which shows that the two estimators cannot suppress the influence of gross errors on reconciled results. The objective function of Welsch [26] estimator is limited, but the bound value is also large, and the influence function does not converge rapidly. Although the influence function of Xie [30] estimator may converge to zero and the convergence speed is improved in comparison to the estimators above, it still cannot achieve the fast standard. Based on the robust estimation theory, a novel robust estimator is proposed to enhance the performance of the robust estimators. The objective function is bounded, the bound value is smaller, and the influence function converges toward zero faster. At the same time, aiming at the problem that the change of feeding composition caused by the long test period of industrial processes is unknown, an iterative robust hierarchical data reconciliation and estimation strategy based on the heat balance is proposed. In order to further verify the robustness and effectiveness of the proposed robust estimator and estimation of feeding composition strategy, a series of comparative experiments are carried out through two numerical examples and the real data from a fluidized bed roaster for zinc smelting.

After initial remarks, motivation aspects and applications of the data reconciliation issue, the paper is organized as follows. Section 1 presents preliminary approaches to traditional data reconciliation and robust estimator. The novel robust estimator is proposed, and two numerical examples are used to demonstrate the effectiveness of the proposed estimator in Section 2. Section 3 provides an iterative robust hierarchical data reconciliation and estimation strategy to estimate feeding composition. The feeding composition of fluidized bed roaster is then estimated in Section 4. Finally, Section 5 provides the conclusions.

## 2. Preliminaries

### 2.1. Data Reconciliation

The purpose of data reconciliation is to obtain the reconciled data by processing the raw measurements. The reconciled data not only satisfy the constraints of the process model but are also closer to the true value. There are three main assumptions for the system using data reconciliation: The system is in a steady state, the measurement errors follow a normal distribution with zero mean, and each measured variable is independent of each other. Taking into account the presence of measurement errors, the measurement model may be expressed as follows:

(1) $$x=x^{*}+\varepsilon$$

where x denotes the vector of raw process measurements, $x^{*}$ denotes the vector of true values of the process variables and ε is the random measurement errors.

Based on the principle of data reconciliation, the general steady-state data reconciliation problem can be stated as a form of solving the weighted least squares solution satisfying the process model and boundary constraints:

(2) $$\begin{array}{l} \min\;{\left(x-\tilde{x}\right)}^{T}\sum^{-1}(x-\tilde{x})\\ s.t.\;\;\;F(\tilde{x},u)=0\\ \;\;\;\;\;\;\;\;\;G(\tilde{x},u)\leq 0 \end{array}$$

where $\tilde{x}$ is the vector of reconciled data, ∑ is the diagonal covariance matrix of measurement errors, u is the vector of unmeasured variables; F represents the process model, which is used as equality constraints in the optimization problem, and G denotes the inequality constraints indicating variable boundaries.

### 2.2. M-Estimator

In the classical weighted least square of data reconciliation, Equation (2), it is usually assumed that the process measurements contain only random errors. However, in the actual measurement process, the measured values may contain gross errors. The presence of gross errors has a serious effect on the conventional data reconciliation, resulting in the propagation of gross errors. This will cause the reconciled data to not satisfy the process model and deviate from the true value of variables seriously. As a result, the robust estimator is used to account for gross errors in measurements.

Currently, there are numerous approaches for robust data reconciliation, the majority of which are based on the theory of M-estimator. The function of the measurement residuals is constructed as the objective function of the robust estimator. By reducing the weight of the measurements with gross errors, it can prevent gross errors smearing other measurements. Hence, the robust estimator can well reconcile the data with gross errors. The problem of robust data reconciliation can be expressed as follows:

(3) $$\begin{array}{l} \min\;\sum_{i=1}^{n}\rho (\xi_{i})=\min\;\sum_{i=1}^{n}\rho (\frac{x_{i}-{\tilde{x}}_{i}}{\sigma_{i}})\\ s.t.\;\;\;F(\tilde{x},u)=0\\ \;\;\;\;\;\;\;\;{\;\tilde{x}}_{i\min}\leq {\tilde{x}}_{i}\leq {\tilde{x}}_{i\max},\;i=1,2,\ldots ,n\\ \;\;\;\;\;\;\;\;{\;u}_{l\min}\leq u_{l}\leq u_{l\max},\;l=1,2,\ldots ,N-n \end{array}$$

where ρ is the robust estimator, $\xi_{i}=\left(x_{i}-{\tilde{x}}_{i}\right)/\sigma_{i}$ is the standardized residual for the ith measured variable, $x_{i},{\;\tilde{x}}_{i}$ and $u_{l}$ are, respectively, the measured data, the reconciled data for the ith measured variable and the estimated value for the jth unmeasured variable, ${\tilde{x}}_{i\min},\;{\tilde{x}}_{i\max},\;u_{l\min}$ and $u_{l\max}$ are, respectively, the lower limit, the upper limit for the ith reconciled variable, the lower limit, the upper limit for the lth unmeasured variable. N, n, N−n denote the total number of variables, the total number of measured variables and the total number of variables not measured, respectively. $\sigma_{i}$ represents the standard deviation for variable measurement errors.

Most M-estimators are not based on a clearly defined probability distribution known in advance. Most of them are based on a simple mathematical structure. The mathematical structure of M-estimator, i.e., ρ, has some general characteristics as follows:

• ρ is is continuous;
• ρ(ξ)=ρ(−ξ);
• ρ(ξ)≥0 and ρ is integrable
• $\rho (\xi_{1})\leq \rho (\xi_{2})$, for $\left|\xi_{i}\right|<\left|\xi_{j}\right|$;
• ρ(0)=0.

As a crucial index in assessing the robustness of the M-estimator, the influence function (IF) [34] is directly proportional to the derivative of the objective function of the estimator. The influence function value refers to the effect of different deviation on the estimator. In general, the function may be defined as the first derivative of the objective function to standardized residuals, which can be expressed as follows:

(4) $$\mathrm{IF}(\xi )=\frac{d\rho (\xi )}{d\xi }$$

Some general characteristics of IF [35] are:

• IF is limited;
• IF is continuous or piecewise continuous;
• IF(−ξ)=−IF(ξ);
• IF is nearly linear near the origin (IF(ξ)≈k⋅ξ,k≠0, for small ξ), but this characteristic is not necessary;
• the rejection point of IF (the point where IF is zero) is finite to suppress large deviations.

On the basis of a robust M-estimation theory, the influence function needs to be continuous and bounded. When ξ is small, the influence function is proportional to ξ; when ξ is infinite, the influence function can converge to a constant, which indicates that the robust estimator can suppress the effect of gross errors in the reconciled results. The influence function of the weighted least squares estimator is the standardized residual ξ, which indicates that the influence function will increase with the measurement errors. It can be seen that the weighted least squares estimator is not robust because its influence function is boundless, and the reconciled results can be easily affected by gross errors.

## 3. Data Reconciliation Based on a Novel Robust Estimator

### 3.1. A Novel Robust Estimator

The most robust estimators are constructed according to the following requirements: The objective function of the estimator is bounded, and the bound value is small; the influence function can rapidly converge to a constant. From the observation of the mathematical structure of 48 different robust estimators in the literature [31], it has been shown that the objective functions of these M-estimators comprise limitless function, limited function, non-piecewise function, piecewise function, multi parameters and a single parameter. The performance of the M-estimator whose objective function is limited, non-piecewise and has single parameter is analyzed. It is found that the M-estimator with a relatively rapid convergence rate basically contains the mathematical expression with e as the base number. The derivative of exponential function based on e is equal to itself. Moreover, $e^{-\xi }$ decreases with increasing ξ and tends towards 0. When *ξ* tends towards 0, $e^{-\xi }$ tends towards 1. Therefore, the objective function can be limited if the structure of $1-e^{-\xi }$ is included in. For instance, the Welsch estimator and the objective function is presented in Equation (5). In order to improve the robustness of the M-estimator, Xie divided $1-e^{-\xi }$ with $1+e^{-\xi }$. By the decline of $1+e^{-\xi }$, the overall rise speed of the objective function is improved. Furthermore, the converging speed of the influence function is accelerated. The objective function of Xie estimator is illustrated under Equation (6).

Welsch:

(5) $$\rho (\xi_{i})=\frac{c_{w}^{2}}{2}\left(1-\exp\left(-{\left(\frac{\xi_{i}}{c_{w}}\right)}^{2}\right)\right)$$

Xie:

(6) $$\rho (\xi_{i})=\frac{1-\exp\left(-{\left(\frac{\xi_{i}}{c_{x}}\right)}^{2}\right)}{1+\exp\left(-{\left(\frac{\xi_{i}}{c_{x}}\right)}^{2}\right)}$$

where $c_{w}$ and $c_{x}$ are, respectively, tuning parameters of Welsch and Xie estimators.

As discussed above, in order to limit the effect of gross errors on the reconciled results, a novel robust estimator is created based on the general characteristics of the M-estimator and influence function. The objective function of the novel robust estimator is defined as follows:

(7) $$\rho (\xi_{i})=\frac{c_{p}^{2}\left[1-\exp\left(-{\left(\xi_{i}/c_{p}\right)}^{2}\right)\right]}{4\left[1+\exp\left(-{\left(\xi_{i}/c_{p}\right)}^{4}\right)\right]}$$

where $c_{p}$ is the tuning parameter; the definition of $\xi_{i}$ is the same as above. By increasing the index of ξ, the downward velocity of $1+e^{-\xi }$ is increased. Then, the objective function increases faster, and the influence function converts to 0 faster. At the same time, in order to satisfy the characteristic that the influence function is almost linear near the origin, the objective function is multiplied by $c_{p}^{2}/4$. The influence function of the novel robust estimator function expressed in Equation (7) is as follows:

(8) $$\mathrm{IF}(\xi_{i})=\frac{d\rho (\xi_{i})}{d\xi_{i}}=\frac{\xi_{i}\exp\left(-{\left(\frac{\xi_{i}}{c_{p}}\right)}^{2}\right)\left[1+\exp\left(-{\left(\frac{\xi_{i}}{c_{p}}\right)}^{4}\right)+\frac{2\xi_{i}^{2}}{c_{p}^{2}}\left(1-\exp\left(-{\left(\frac{\xi_{i}}{c_{p}}\right)}^{2}\right)\right)\right]}{2{\left[1+\exp\left(-{\left(\frac{\xi_{i}}{c_{p}}\right)}^{4}\right)\right]}^{2}}$$

It can be seen from Equations (7) and (8) that when the standardized residual is small, the objective function of the novel robust estimator tends to be gradually zero, and the influence function is proportional to the standardized residual. It shows that the weighted least squares method can be used to reconcile the data where measurements contain only random errors. When the standardized residual is significant, the objective function of the novel robust estimator tends to be constant little by little, and the influence function quickly converges to zero. It is demonstrated that the novel robust estimator can adjust the weight of the estimator based on the standardized residual size when the measurements contain gross errors. The larger the gross errors, the lower the weights, and the greater the effects of restraining the gross errors. Therefore, the proposed estimator is more robust.

### 3.2. The Tuning Parameter of the Novel Robust Estimator

The use of robust estimators in data regression typically involves the inverse relationship between relative efficiency and robustness [36]. Relative efficiency refers to the fit quality of the estimated value reconciled by M-estimator, when errors follow another distribution relative to the reference distribution (usually assumed to be Normal distribution). The so-called robustness refers to the performance of the M-estimator under various nonnormal error distributions. The more robust an M-estimator is, the less its relative efficiency is. The relationship between robustness and relative efficiency is addressed by the tuning parameters. When comparing the performance of different M-estimators, the level of relative efficiency and the reference distribution should be consistent. In general, the tuning parameters for each M-estimator are obtained at the relative efficiency level of 95%. Then the robustness of distinct M-estimators is judged.

The mathematical definition of relative efficiency is illustrated in Equation (9):

(9) $$E_{ff}[\mathrm{IF}(\xi ),f(\xi )]=\frac{V_{f}[{\mathrm{IF}}_{f}(\xi ),f(\xi )]}{V[\mathrm{IF}(\xi ),f(\xi )]}$$

where $V_{f}$ is the asymptotic variance of the reference estimator, V is the asymptotic variance of the specified estimator, f(ξ) is the error reference probability distribution, ${\mathrm{IF}}_{f}(\xi )$ is the influence function of the reference estimator and IF(ξ) is the influence function of the specified estimator. The asymptotic variance V is defined as follows [37]:

(10) $$V[\mathrm{IF}(\xi ),f(\xi )]=\frac{\int_{-\infty }^{+\infty }{\mathrm{IF}}^{2}(\xi )f(\xi )d\xi }{{\left[\int_{-\infty }^{+\infty }{\mathrm{IF}}^{\prime }(\xi )f(\xi )d\xi \right]}^{2}}$$

where ${\mathrm{IF}}^{\prime }(\xi )$ is the derivative of the influence function IF(ξ). Since ${\mathrm{IF}}^{\prime }(\xi )$ may be discontinuous, Equation (10) can be further expressed by Equation (11):

(11) $$V[\mathrm{IF}(\xi ),f(\xi )]=\frac{\int_{-\infty }^{+\infty }{\mathrm{IF}}^{2}(\xi )f(\xi )d\xi }{{\left[-\int_{-\infty }^{+\infty }\mathrm{IF}(\xi )f^{\prime }(\xi )d\xi \right]}^{2}}=\frac{\int_{-\infty }^{+\infty }{\mathrm{IF}}^{2}(\xi )f(\xi )d\xi }{{\left[-\int_{-\infty }^{+\infty }\mathrm{IF}(\xi )f^{\prime }(\xi )d\xi \right]}^{2}}$$

where $f^{\prime }(\xi )$ is the derivative of f(ξ).

The tuning parameter of the proposed estimator at the relative efficiency level of 95% in respect to the Normal distribution is calculated as follows:

(1) The Normal distribution is selected as the reference distribution, and its probabilistic distribution and the first derivative are respectively expressed as follows:

(12) $$f(\xi )=\frac{1}{\sigma \sqrt{2\pi }}\exp\left(-\frac{\xi^{2}}{2}\right)$$

(13) $$f^{\prime }(\xi )=-\xi \frac{1}{\sigma \sqrt{2\pi }}\exp\left(-\frac{\xi^{2}}{2}\right)=-\xi f(\xi )$$

(2) The least square estimator is selected as the reference estimator, and its influence function is expressed as follows:

(14) $${\mathrm{IF}}_{f}(\xi )=\frac{d\rho (\xi )}{d\xi }=\frac{d}{d\xi }\left(\frac{\xi^{2}}{2}\right)=\xi$$

(3) The influence function of the proposed estimator is shown in Equation (8). The expressions of $V_{f}$ and V can be obtained by using Equation (11):

(15) $$V_{f}=\frac{2\cdot \int_{0}^{+\infty }{\mathrm{IF}}_{f}^{2}(\xi )f(\xi )d\xi }{{\left(2\cdot \int_{0}^{+\infty }{\mathrm{IF}}_{f}(\xi )\xi f(\xi )d\xi \right)}^{2}}$$

(16) $$V=\frac{2\cdot \int_{0}^{+\infty }{\mathrm{IF}}^{2}(\xi )f(\xi )d\xi }{{\left(2\cdot \int_{0}^{+\infty }\mathrm{IF}(\xi )\xi f(\xi )d\xi \right)}^{2}}$$

(4) The expression of relative efficiency can be obtained from Equation (9). Making the expression equal to 0.95, a univariate equation on the tuning parameter $c_{p}$ can be constructed, as shown in Equation (17). By numerical calculation, the tuning parameter at the relative efficiency level of 95% in respect to the Normal distribution is 1.5424.

(17) $$\begin{array}{l} \frac{V_{f}}{V}=\\ \frac{\frac{2\int_{0}^{+\infty }\xi^{2}\cdot \frac{1}{\sigma \sqrt{2\pi }}\exp(-(\xi^{2}/2))d\xi }{{\left[2\int_{0}^{+\infty }\xi^{2}\cdot \frac{1}{\sigma \sqrt{2\pi }}\exp(-(\xi^{2}/2))d\xi \right]}^{2}}}{\frac{2\int_{0}^{+\infty }\frac{\xi^{2}\exp(-2{\left(\xi /c_{p}\right)}^{2}){\left[1+\exp(-{\left(\xi /c_{p}\right)}^{4})+2\xi^{2}(1-\exp(-{\left(\xi /c_{p}\right)}^{2}))/c_{p}^{2}\right]}^{2}}{4[1+\exp{\left(-{\left(\xi /c_{p}\right)}^{4}\right]}^{4}}\cdot \frac{1}{\sigma \sqrt{2\pi }}\exp(-(\xi^{2}/2))d\xi }{{\left[2\int_{0}^{+\infty }\frac{\xi^{2}\exp(-{\left(\xi /c_{p}\right)}^{2})[1+\exp(-{\left(\xi /c_{p}\right)}^{4})+2\xi^{2}(1-\exp(-{\left(\xi /c_{p}\right)}^{2}))/c_{p}^{2}]}{2[1+\exp{\left(-{\left(\xi /c_{p}\right)}^{4}\right]}^{2}}\cdot \frac{1}{\sigma \sqrt{2\pi }}\exp(-(\xi^{2}/2))d\xi \right]}^{2}}} \end{array}=0.95$$

**Figure 1:**
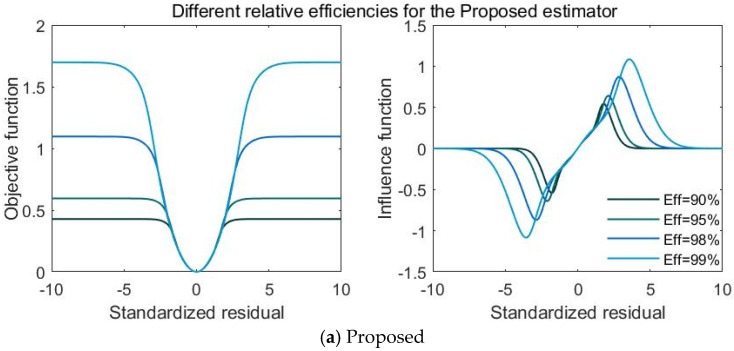

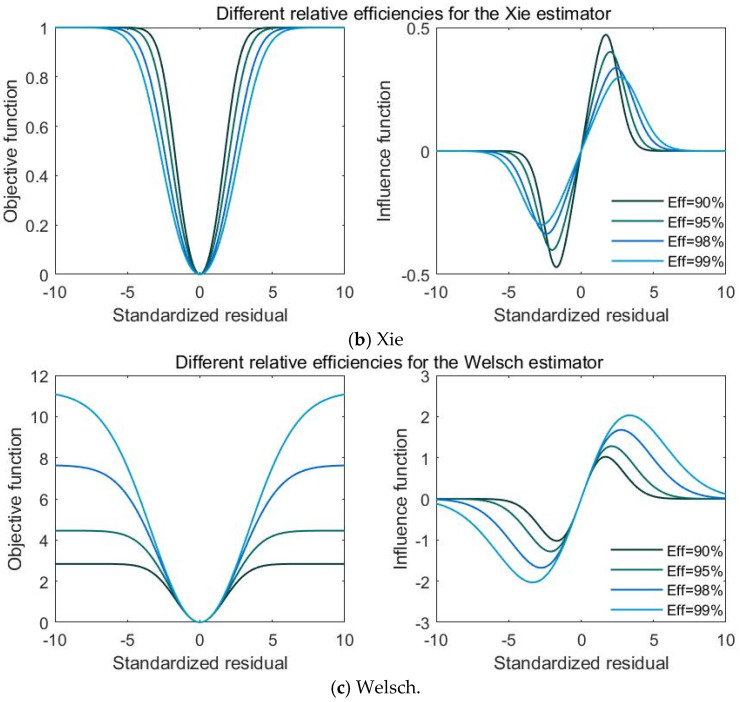
Characteristicfunctions of three estimators at distinct relative efficiency levels.

To more accurately reflect the competitive relationship between the relative efficiency and robustness of the M-estimator, the comparative images of the objective function and influence function of the proposed, Xie and Welsch estimators are shown in Figure 1. Relative efficiency levels are 90%, 95%, 98% and 99%, respectively. The tuning parameters of the three estimators at different relative efficiency levels are presented in Table 1. Table 1 and Figure 1 show that the higher the relative efficiency level, the higher the values of the tuning parameters for the three estimators. The higher the tuning parameter, the slower the convergence speed of the influence function to 0 and the weaker the robustness. Hence, when comparing the robustness of distinct estimators, the comparative analysis has to be performed at the same level of relative efficiency.

In order to further analyze the effectiveness of the proposed estimator, the objective function and influence function of the proposed estimator are compared with the other four robust estimators. The four types of robust estimators are Fair, Cauchy, Welsch and Xie. The objective functions of Fair and Cauchy are shown in Equations (18) and (19):

Fair:

(18) $$\rho (\xi_{i})=c_{f}^{2}\left(\frac{\left|\xi_{i}\right|}{c_{f}}-\ln\left(1+\frac{\left|\xi_{i}\right|}{c_{f}}\right)\right)$$

**Figure 2:**
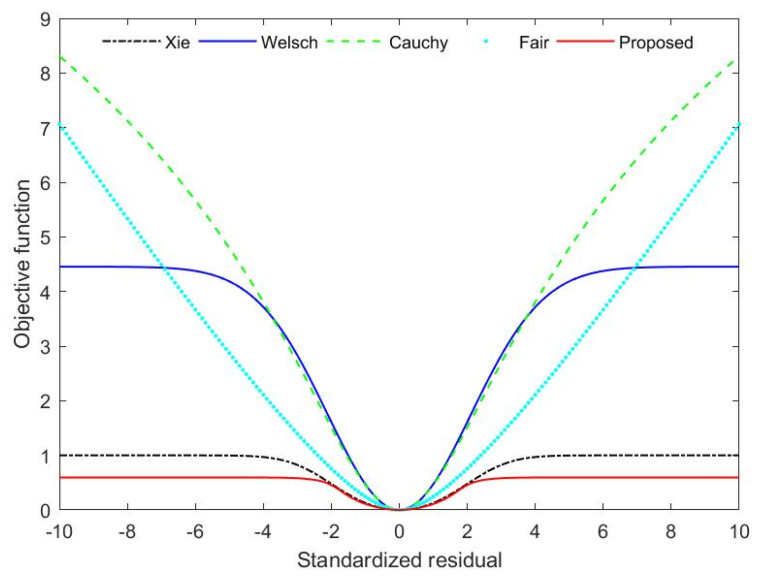
Objective functions for Xie, Welsch, Cauchy, Fair and Proposed estimator.

**Figure 3:**
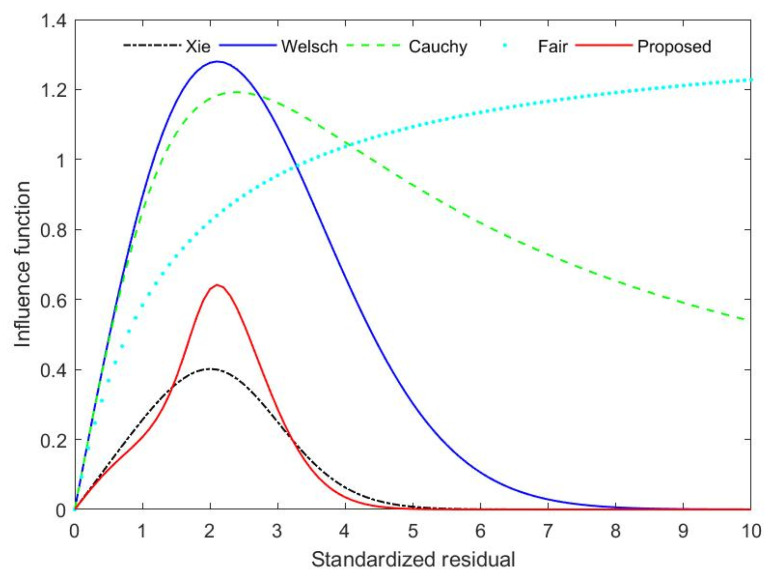
Influence functions for Xie, Welsch, Cauchy, Fair and Proposed estimator.

Cauchy:

(19) $$\rho (\xi_{i})=c_{c}^{2}\ln\left(1+\frac{\xi_{i}^{2}}{c_{c}^{2}}\right)$$

where $c_{f}$ and $c_{c}$ are, respectively, tuning parameters of Fair and Cauchy estimators. The tuning parameters of the five M-estimators under the level of 95% relative efficiency are $c_{f}=1.3998$, $c_{w}=2.9846$, $c_{c}=2.3849$, $c_{x}=1.9597$ and $c_{p}=1.5424$. The results of the comparison are illustrated in Figure 2 and Figure 3.

As can be seen from Figure 2 and Figure 3, the objective function and influence function of the proposed M-estimator satisfy their general characteristics. When the standardized residual is low, the objective and influence functions of several robust estimators are all close, which indicates that low measurement errors have little effect on these estimators. When the standardized residual is large, the objective function of the Fair and Cauchy estimator increases at a higher rate. Their influence functions do not converge rapidly and eventually converge to a nonzero value. It shows that both Fair and Cauchy estimators are sensitive to gross errors. Although the objective function of Welsch does not diverge, it tends to be a greater constant than Xie and the novel robust estimator. The objective functions of the novel robust estimator and Xie estimator are less than those of the other three estimators in the case of gross errors in measurements. Furthermore, the proposed M-estimator tends to be a constant lower than Xie. It can be seen further from Figure 2 that when there are gross errors in measurements, the influence function of the proposed M-estimator decreases much faster than that of Xie. When the standardized residual exceeds 4, the influence function of the proposed M-estimator converges to 0. Nevertheless, the influence function of Xie converges to 0 only when the standardized residual is above 5. Hence one can see that the convergence speed of influence function of the novel robust estimator is faster than that of Xie, and the effect of suppressing gross errors is excellent. The proposed method is therefore more robust and efficient than other estimators.

Next, two numerical examples are used to further verify the effectiveness of the proposed robust estimator, and the proposed robust estimator is compared with several estimators. The data reconciliation procedure can be interpreted mathematically as an optimization problem. In this paper, the state transition algorithm (STA) is applied as the optimization method for data reconciliation problems [38]. The local search operator, the global search operator and the heuristic search operator are adopted as the state transformation operators. This can prevent the solution process from falling into the local optimum and reduce the search time for the global optimum.

### 3.3. Linear Case

**Figure 4:**
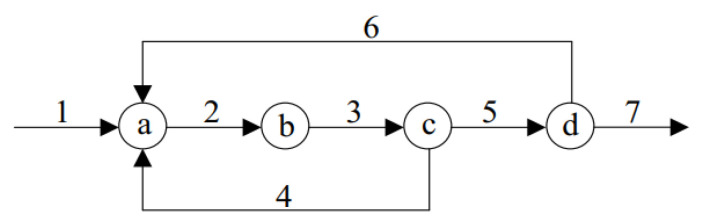
Diagram of the measurement network.

In this part, the measurement network [26] as shown in Figure 4 is adopted. The network is a linear structure consisting of four nodes and seven streams. As can be seen from Figure 4, the seven streams meet the following material balance

(20) $$\begin{array}{l} x_{1}-x_{2}+x_{4}+x_{6}=0\\ x_{2}-x_{3}=0\\ x_{3}-x_{4}-x_{5}=0\\ x_{5}-x_{6}-x_{7}=0 \end{array}$$

where $x={\left[x_{1},{\;x}_{2},\;\ldots \;,{\;x}_{7}\right]}^{T}$ is set of stream variables. In this linear example, all variables are assumed to be measured, and the true value of each variable is X = [5, 15, 15, 5, 10, 5, 5]. The true value of each variable is added with some random errors to get the corresponding measurements. Let the standard deviation of random errors be 2.5% of the true value of each stream. The diagonal matrix of the variance is as follows:

(21) ∑=0.01×diag(1.562,14.062,14.062,1.562,6.250,1.562,1.562)

For a precise analysis of the performance of each robust estimator, SSE (sum of squares due to error), TER (total error reduction) and RER (relative error reduction) [27] are used as indicators to assess the precision of the reconciled results. The definitions included are:

(22) $$\mathrm{SSE}=\sum_{i=1}^{n}{\left({\tilde{x}}_{i}-x_{i}^{*}\right)}^{2}+\sum_{j=1}^{m}{\left({\tilde{u}}_{j}-u_{j}^{*}\right)}^{2}$$

(23) $$\mathrm{TER}=\frac{\sqrt{\sum_{i=1}^{n}{\left(\frac{x_{i}-x_{i}^{*}}{\sigma_{i}}\right)}^{2}}-\sqrt{\sum_{i=1}^{n}{\left(\frac{{\tilde{x}}_{i}-x_{i}^{*}}{\sigma_{i}}\right)}^{2}}}{\sqrt{\sum_{i=1}^{n}{\left(\frac{x_{i}-x_{i}^{*}}{\sigma_{i}}\right)}^{2}}}$$

(24) $$\mathrm{RER}=\frac{\sum_{i=1}^{n}\left(\frac{\left|x_{i}^{*}-x_{i}\right|}{x_{i}^{*}}-\frac{\left|x_{i}^{*}-{\tilde{x}}_{i}\right|}{x_{i}^{*}}\right)}{\sum_{i=1}^{n}\frac{\left|x_{i}^{*}-x_{i}\right|}{x_{i}^{*}}}$$

where ${\tilde{x}}_{i}$ represents the reconciled data for the ith measurable variable; $x_{i}^{*}$ represents the true value for the ith measurable variable; $x_{i}$ represents the measured data for the ith measurable variable; ${\tilde{u}}_{j}$ is the estimated value of the jth unmeasurable variable; $u_{j}^{*}$ is the true value of the jth unmeasurable variable; $\sigma_{i}$ denotes the standard deviation for the ith measurable variable; n is the total number of measurable variables; m is the total number of unmeasurable variables. The smaller the SSE, the closer the reconciled results are to the true value. The larger the TER, the more accurate the reconciled results. The larger RER shows that the reconciled results are less affected by gross errors.

#### 3.3.1. There Are Two Gross Errors in the Measurement Variables

Streams 2 and 5 are selected to add gross errors in the magnitude of 2 and 1, respectively, and other streams contain only random errors. The results of the data reconciliation and indicators for several methods are presented in Table 2. For convenience, "Proposed" is used to represent the proposed estimator in this table and subsequent tables. 

Table 2 shows that the SSE of the proposed robust estimator (Proposed) is much lower than that of Fair with 1.2117 and Cauchy with 0.1287 and is less than that of Xie and Welsch estimator. The TER of the novel robust estimator is much larger than that of Fair with 0.2953 and Cauchy with 0.7606, and higher than that of Xie and Welsch estimator. In addition, the RER of Proposed is also higher than that of the other four robust estimators. The results show that in the case of two gross errors, the reconciled results of the novel robust estimator are closer to the true value, and the proposed estimator performs better than other methods.

#### 3.3.2. There Are Three Gross Errors in the Measurement Variables

Streams 2, 5 and 7 are selected to add gross errors with the magnitudes of 2, 1 and −1.5, respectively, and other streams contain only random errors. The results of the data reconciliation and indicators for several methods are presented in Table 3.

Table 3 shows that the SSE of the proposed robust estimator (Proposed) is much lower than that of Fair with 1.2866 and Cauchy with 0.1071 and is less than that of Xie and Welsch estimator. The TER of the novel robust estimator is much larger than that of Fair with 0.3474, and higher than that of Xie, Welsch and Cauchy estimator. In addition, the RER of Proposed is also higher than that of the other four robust estimators. The results show that in the case of three gross errors, the reconciled results of the novel robust estimator are closer to the true value, and the proposed estimator performs better than other methods.

In order to better illustrate the effectiveness of the proposed estimator, the linear numerical example is divided into three groups for comparative experiments. Each group comprises 100 data samples. In the first group, there is gross error in stream 2 ($x_{2}$). In group two, there are gross errors in stream 2 ($x_{2}$) and stream 5 ($x_{5}$). The third group contains gross errors in stream 2 ($x_{2}$), stream 5 ($x_{5}$) and stream 7 ($x_{7}$). In each group of experiments, the selected variables are added with gross errors ranging from 3 times to 9 times the standard deviation. The measurement test (MT) is used to detect gross errors. Two criteria are selected to assess the performance of different methods, including the observed power (*OP*) and the Average Number of Type I errors (*ATVI*) [2]. *OP* and *ATVI* are defined as follows:

(25) $$OP=\frac{\mathrm{The}\;\mathrm{number}\;\mathrm{of}\;\mathrm{gross}\;\mathrm{errors}\;\mathrm{detected}\;\mathrm{correctly}}{\mathrm{The}\;\mathrm{total}\;\mathrm{number}\;\mathrm{of}\;\mathrm{gross}\;\mathrm{errors}}$$

(26) $$ATVI=\frac{\mathrm{The}\;\mathrm{number}\;\mathrm{of}\;\mathrm{gross}\;\mathrm{errors}\;\mathrm{detected}\;\mathrm{mistakenly}}{\mathrm{The}\;\mathrm{total}\;\mathrm{number}\;\mathrm{of}\;\mathrm{simulations}}$$

**Figure 5:**
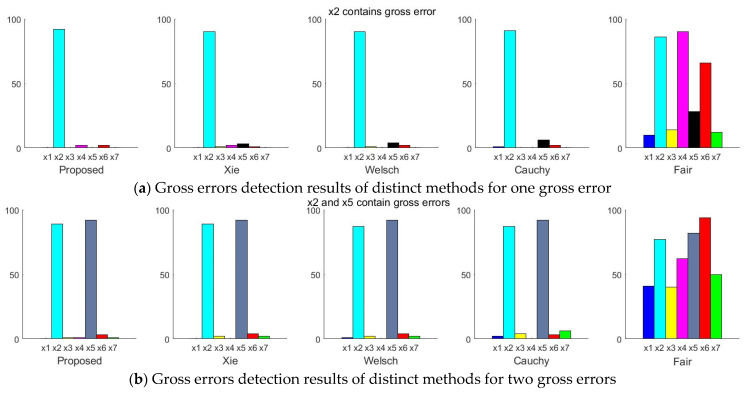

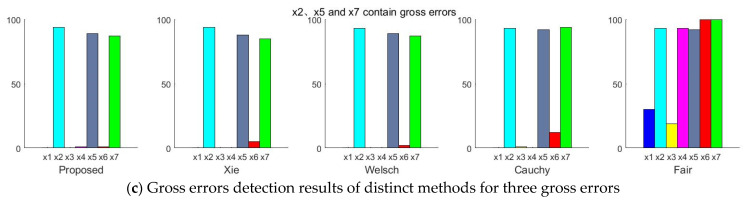
Statistical diagram of gross errors detection process for distinct methods.

**Figure 6:**
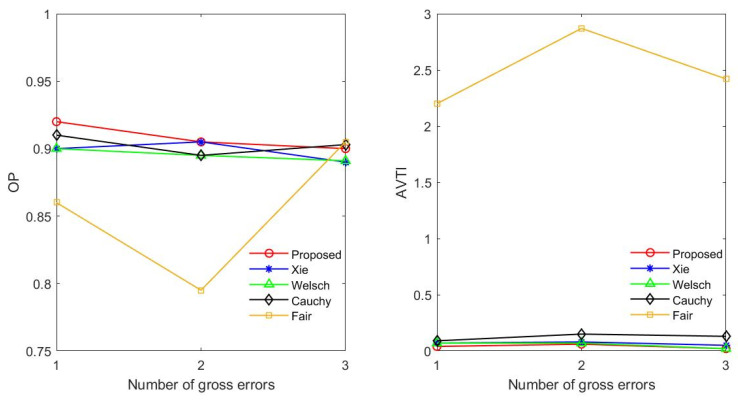
Results of *OP* and *AVTI* with different methods.

*OP* indicates the number of gross errors correctly detected. *ATVI* indicates the number of gross errors mistakenly detected. The larger value of the *OP* is, the stronger ability of the estimator to identify gross errors. The lower value of the *ATVI* is, the less frequently gross errors are detected incorrectly. The statistical graph of the process in which gross errors are detected by distinct methods in each group is shown in Figure 5. The abscissa of each graph corresponds to seven stream variables. The y-axis is the number of times each variable is detected to contain gross errors. The *OP* and *ATVI* of the proposed estimator, Xie, Welsch, Cauchy and Fair are obtained by statistical analysis of the process diagram, as illustrated in Figure 6.

As depicted in Figure 6, when one variable, two variables and three variables contain gross errors, the *OP* obtained by the proposed estimator is higher than that obtained by other estimators. The result shows that the proposed estimator has a higher probability of identifying gross errors correctly. In addition, the *AVTI* obtained by the proposed estimator is lower than that obtained by other methods. It can also be seen from Figure 5 that the proposed estimator has a lower number of gross errors mistakenly detected than other estimators. It is shown that gross errors have less influence on the data reconciliation based on the Proposed estimator.

### 3.4. Nonlinear Case

A nonlinear numerical example [22] is used in this part. Within the nonlinear system, there are five measurable variables, three unmeasurable variables and six nonlinear constraint equations, which are described as Equation (27):

(27) $$\left\{ \begin{array}{l} 0.5x_{1}^{2}-0.7x_{2}+x_{3}u_{1}+x_{2}^{2}u_{1}u_{2}+2x_{3}u_{3}^{2}-255.8=0\\ x_{1}-2x_{2}+3x_{1}x_{3}-2x_{2}u_{1}-x_{2}u_{2}u_{3}+111.2=0\\ x_{3}u_{1}-x_{1}+3x_{2}+x_{1}u_{2}-x_{3}\sqrt{u_{3}}-33.57=0\\ x_{4}-x_{1}-x_{3}^{2}+u_{2}+3u_{3}=0\\ x_{5}-2x_{3}u_{2}u_{3}=0\\ 2x_{1}+x_{2}x_{3}u_{1}+u_{2}-u_{3}-126.6=0 \end{array}\right.$$

where $x_{i}(i=1,2,\ldots ,5)$ is the measured variable and $u_{j}(j=1,2,3)$ is the unmeasured variable. Measurements of the measured variables are obtained from the true value added, some random errors and gross errors. The true value, the measured value, the standard deviation of the measured variables and the true value of the unmeasured variables are given in Table 4. Amongst these, $\;x_{2}\;$,$\;x_{3}$ and $x_{5}$ contain gross errors. The results of the data reconciliation and indicators for several methods are presented in Table 4.

Table 4 shows that the SSE calculated by the proposed robust estimator is significantly less than that calculated by Fair, Cauchy and Welsch estimator, and it is less than that calculated by Xie estimator. Furthermore, the TER and RER of the proposed estimator are larger than those of the other four estimators. The results show tha for the nonlinear case, the reconciled results of the new robust estimator are also near the true value. Consequently, the proposed robust estimator can effectively suppress the effect of gross errors on the reconciled results and has greater robustness and excellent effectiveness.

## 4. Feeding Composition Estimation Based on Iterative Data Reconciliation

In order to address the problem caused by the unknown change in feeding composition, an iterative robust hierarchical data reconciliation and estimation strategy is proposed. The method is applied to a fluidized bed roaster for zinc smelting. Firstly, the industrial process of fluidized bed roaster is introduced. At the same time, difficulties in reconciling the fluidized bed roaster are discussed in Section 3.1. Then, on the basis of the establishment of the mechanism balance model of the fluidized bed roaster, specific solutions are presented in accordance with the existing difficulties.

### 4.1. Industrial Process Description

**Figure 7:**
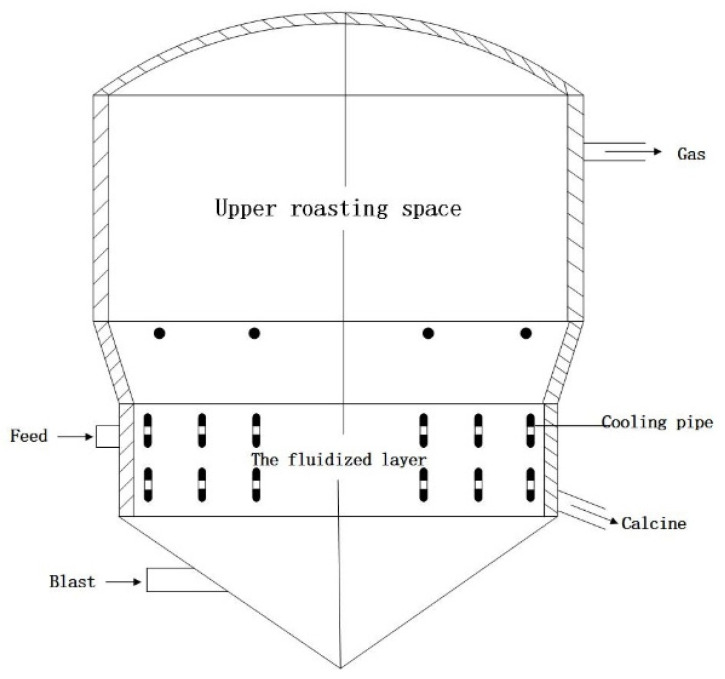
Structure diagram of fluidized bed roaster.

The fluidized bed roaster is a type of thermal equipment that applies fluidization technology to make materials desulphurized by oxidation roasting in the metallurgical industry. The roasting process of the fluidized bed roaster is a gas-solid reaction procedure. By sending a great deal of air into the oven from the bottom, a very intense exothermic reaction occurs in the material layer of the sulfur ore under the agitation of the air. Oxygen combines with sulfide to form sulfur dioxide and precious metals are converted into oxides or sulphates. The structure diagram for the fluidized bed roaster is shown in Figure 7.

In the fluidized roasting process, accurate and reliable measurements are the basis of process control, functional analysis and production management. However, in the real process, due to inaccurate sensors, equipment leaks and measurement bias, real-time measurements are inevitably affected by random errors and gross errors. That has a serious effect on the accuracy of measurements. In addition, some important variables, such as smoke volume and calcine quality, cannot be measured due to limitations in measurement technology and the environment.

The ZnS content of the feed during the roasting of the zinc concentrate will affect the indices of operation, such as the resistance to roasting, the temperature of the boiling layer and the content in the gaseous effluents. Then, the technical-economic index of the level of zinc soluble in calcine is affected, which field staff can judge from according to roasting. The compositions of mixed zinc concentrate and calcine are sampled only twice a day, once in the morning and once at night. Then the percentage of Zn, S and other elements in the mixed zinc concentrate and that of soluble zinc and soluble sulfur in the calcine are detected by the laboratory. Nevertheless, the sampling frequency of the variables measured in real time, like feed volume and blast volume, is 5 seconds, which is much greater than that of the laboratory data. Hence, due to the long test cycle of the mixed zinc concentrate composition in the fluidized bed roaster, the laboratory data cannot reflect the change in the real-time feeding composition.

Concerning the problems of measurements contaminated by gross errors, irredundant process data and a long test cycle of the feeding composition, an iterative robust hierarchical data reconciliation and estimation strategy for feeding composition is proposed.

### 4.2. Iterative Robust Hierarchical Data Reconciliation and Composition Estimation Framework

**Figure 8:**
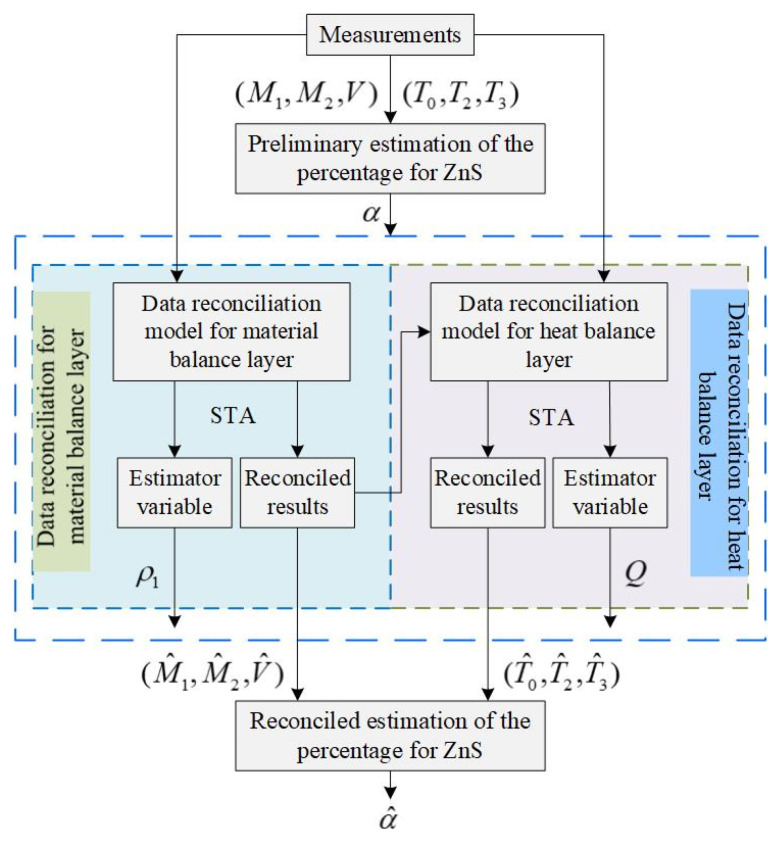
The framework of iterative robust hierarchical data reconciliation and composition estimation.

Figure 8 illustrates the framework of iterative robust hierarchical data reconciliation and estimation strategy for feeding composition. The major steps are as follows:

Step 1: Establish the steady-state mechanism model of the fluidized bed roaster The establishment of a reasonable process model is the basis for data reconciliation. However, the actual industrial process is complex and needs to be simplified accordingly. The following assumptions are put forward to establish a credible and comprehensible mechanism model:

(1) The only exothermal component of the mixed zinc concentrate is ZnS.

(2) Since the specific heat of calcine and soot is close and the mass ratio is close to 1:1, soot can be classified as calcine in the calculation.

Therefore, the material and heat balance at steady-state are expressed by Equations (28) and (29):

Material balance:

(28) $$\frac{16}{97}\alpha (M_{1}+M_{2})1000+V_{0}(\rho_{0}-\rho_{1})=0$$

Heat balance:

(29) $$\begin{array}{l} 1000(M_{1}+M_{2})T_{1}cp_{1}+V_{0}T_{0}cp_{2}+\frac{443508}{97}\alpha (M_{1}+M_{2})1000\\ =1000(1-\frac{16}{97}\alpha )(M_{1}+M_{2})T_{3}cp_{3}+V_{0}T_{2}cp_{0}+Q \end{array}$$

where α denotes the percentage of ZnS in zinc concentrate; $M_{1}$ and $M_{2}$ represent the first and second feeding quantity, respectively; $V_{0}$ is the blast velocity; $T_{0}$, $T_{1}$, $T_{2}$ and $T_{3}$ denote the temperature variables for blast, zinc concentrate, gas and calcine, respectively; $cp_{0}$, $cp_{1}$, $cp_{2}$ and $cp_{3}$ are specific heat for gas, zinc concentrate, blast and calcine, respectively; *Q* describe the heat loss; $\rho_{0}$ and $\rho_{1}$ are, respectively, the density of air and gas. Among these variables, $M_{1}$, $M_{2}$, $V_{0}$, $T_{0}$, $T_{2}$ and $T_{3}$ are measured variables. $\rho_{1}$, Q and α are unmeasured variables. The other parameters are all fixed values.

Step 2: Preliminary estimation of α based on measurements and heat balance. Estimation of α can be considered as an optimization problem. Combined with the heat balance and measurements, the ZnS estimation model before data reconciliation could be expressed as follows:

(30) $$\begin{array}{l} \min Y_{1}=(1000(M_{1}+M_{2})T_{1}cp_{1}+V_{0}T_{0}cp_{2}+\frac{443,508}{97}\alpha (M_{1}+M_{2})1000\\ \;\;\;\;-1000(1-\frac{16}{97}\alpha )(M_{1}+M_{2})T_{3}cp_{3}-V_{0}T_{2}cp_{0}-Q{)}^{2} \end{array}$$

(31) $$s.t.\;\;\;\;\;\alpha_{\min}\leq \alpha \leq \alpha_{\max}$$

where $Y_{1}$ is the objective of the optimization problem; $\alpha_{\min}$ and $\alpha_{\max}$ are the top and bottom bounds of α, respectively. In this case, Q can be roughly considered as a fixed value, which can be obtained by expert knowledge. The preliminary estimation of α will be used to construct the hierarchical model in Step 3.

Step 3: Robust hierarchical data reconciliation. First, the robust reconciliation model of material balance layer is established to obtain the reconciled values of the first and second feeding quantity and blast velocity. Then, the reconciled values of the material balance layer are used as precise values to construct the robust reconciliation model of heat balance layer. Finally, the reconciled results of the temperature for the blast, gas and calcine can be obtained. Therefore, the hierarchical data reconciliation model based on the novel robust estimation is expressed as follows:

Material balance layer:

(32) $$\begin{array}{ll} \min f_{1} & =\sum_{i=1}^{n}\rho (x_{i},{\hat{x}}_{i})\\ & =\sum_{i=1}^{n}\left(1+({\left(x_{i}-{\hat{x}}_{i}\right)}^{2}/\sigma_{i}^{2}c_{p}^{14}-1)\exp(-{\left(\left(x_{i}-{\hat{x}}_{i}\right)/\sigma_{i}c_{p}\right)}^{2})\right)\\ & =1+({\left(M_{1}-{\hat{M}}_{1}\right)}^{2}/\sigma_{M_{1}}^{2}c_{p}^{14}-1)\exp(-{\left(\left(M_{1}-{\hat{M}}_{1}\right)/\sigma_{M_{1}}c_{p}\right)}^{2})+\\ & \;1+({\left(M_{2}-{\hat{M}}_{2}\right)}^{2}/\sigma_{M_{2}}^{2}c_{p}^{14}-1)\exp(-{\left(\left(M_{2}-{\hat{M}}_{2}\right)/\sigma_{M_{2}}c_{p}\right)}^{2})+\\ & \;1+({\left(V_{0}-{\hat{V}}_{0}\right)}^{2}/\sigma_{V_{0}}^{2}c_{p}^{14}-1)\exp(-{\left(\left(V_{0}-{\hat{V}}_{0}\right)/\sigma_{V_{0}}c_{p}\right)}^{2})+\\ & \;1+({\left\|\rho_{1}-{\tilde{\rho }}_{1}\right\|}^{2}/c_{p}^{14}-1)\exp(-(\left\|\rho_{1}-{\tilde{\rho }}_{1}\right\|/c_{p}{)}^{2}) \end{array}$$

(33) $$s.t.\;\left\{ \begin{array}{l} \frac{16}{97}\alpha ({\hat{M}}_{1}+{\hat{M}}_{2})+{\hat{V}}_{0}(\rho_{0}-\rho_{1})=0\\ {\hat{M}}_{1\min}\leq {\hat{M}}_{1}\leq {\hat{M}}_{1\max}\\ {\hat{M}}_{2\min}\leq {\hat{M}}_{2}\leq {\hat{M}}_{2\max}\\ {\hat{V}}_{0\min}\leq {\hat{V}}_{0}\leq {\hat{V}}_{0\max}\\ \rho_{1\min}\leq \rho_{1}\leq \rho_{1\max} \end{array}\right.$$

where $f_{1}$ is the objective function of the robust estimator; $x_{i}$ and $\hat{x_{i}}$ represent the measurement and reconciled data for the ith measured variable respectively; ${\hat{M}}_{1\min}$, ${\hat{M}}_{2\min}$, ${\hat{V}}_{0\min}$ and $\rho_{1\min}$ denote the bottom bounds of the reconciled results for the first and second feeding quantity, blast velocity and the density of the air, respectively. ${\hat{M}}_{1\max}$, ${\hat{M}}_{2\max}$, ${\hat{V}}_{0\max}$ and $\rho_{1\max}$ denote the top bounds of the reconciled results for the first and second feeding quantity, blast velocity and the density of the air, respectively.

Heat balance layer:

(34) $$\begin{array}{ll} \min f_{2} & =\sum_{i=1}^{n}\rho (x_{i},{\hat{x}}_{i})\\ & =\sum_{i=1}^{n}(1+({\left(x_{i}-{\hat{x}}_{i}\right)}^{2}/\sigma_{i}^{2}c_{p}^{14}-1)\exp(-\left(\left(x_{i}-{\hat{x}}_{i}\right)/\sigma_{i}c_{p}{)}^{2})\right)\\ & =1+({\left(T_{0}-{\hat{T}}_{0}\right)}^{2}/\sigma_{T_{0}}^{2}c_{p}^{14}-1)\exp(-(\left(T_{0}-{\hat{T}}_{0}\right)/\sigma_{T_{0}}c_{p}{)}^{2})+\\ & \;1+({\left(T_{2}-{\hat{T}}_{2}\right)}^{2}/\sigma_{T_{2}}^{2}c_{p}^{14}-1)\exp(-(\left(T_{2}-{\hat{T}}_{2}\right)/\sigma_{T2}c_{p}{)}^{2})+\\ & \;1+({\left(T_{3}-{\hat{T}}_{3}\right)}^{2}/\sigma_{T_{3}}^{2}c_{p}^{14}-1)\exp(-{\left(\left(T_{3}-{\hat{T}}_{3}\right)/\sigma_{T_{3}}c_{p}\right)}^{2})+\\ & \;1+({\left\|Q-\tilde{Q}\right\|}^{2}/c_{p}^{14}-1)\exp(-(\left\|Q-\tilde{Q}\right\|/c_{p}{)}^{2}) \end{array}$$

(35) $$s.t.\left\{ \begin{array}{l} 1000({\hat{M}}_{1}+{\hat{M}}_{2})T_{1}cp_{1}+{\hat{V}}_{0}{\hat{T}}_{0}cp_{2}+\frac{443508}{97}\alpha ({\hat{M}}_{1}+{\hat{M}}_{2})1000\\ =1000(1-\frac{16}{97}\alpha )({\hat{M}}_{1}+{\hat{M}}_{2}){\hat{T}}_{3}cp_{3}+{\hat{V}}_{0}{\hat{T}}_{2}cp_{0}+Q\\ {\hat{T}}_{0\min}\leq {\hat{T}}_{0}\leq {\hat{T}}_{0\max}\\ {\hat{T}}_{2\min}\leq {\hat{T}}_{2}\leq {\hat{T}}_{2\max}\\ {\hat{T}}_{3\min}\leq {\hat{T}}_{3}\leq {\hat{T}}_{3\max}\\ Q_{\min}\leq Q\leq Q_{\max} \end{array}\right.$$

where $f_{2}$ is the objective function of the robust estimator; ${\hat{T}}_{0\min}$, ${\hat{T}}_{2\min}$, ${\hat{T}}_{3\min}$ and $Q_{\min}$ denote the bottom bounds of the reconciled results for the temperature of the blast, gas, calcine and heat loss, respectively; ${\hat{T}}_{0\max}$, ${\hat{T}}_{2\max}$, ${\hat{T}}_{3\max}$ and $Q_{\max}$ denote the top bounds of the reconciled results for the temperature of the blast, gas, calcine and heat loss, respectively.

Step 4: Reconciled estimation of $\hat{\alpha }$ based on reconciled results and heat balance. The reconciled results obtained in Step 3 are substituted for the objective function of the optimization problem based on heat balance. Then the estimated value of the percentage for ZnS after reconciliation could be obtained. The ZnS estimation model after data reconciliation can be represented as follows:

(36) $$\begin{array}{l} \min Y_{2}=(1000({\hat{M}}_{1}+{\hat{M}}_{2})T_{1}cp_{1}+{\hat{V}}_{0}{\hat{T}}_{0}cp_{2}+\frac{443,508}{97}\hat{\alpha }({\hat{M}}_{1}+{\hat{M}}_{2})1000\\ \;\;\;\;-1000(1-\frac{16}{97}\hat{\alpha })({\hat{M}}_{1}+{\hat{M}}_{2}){\hat{T}}_{3}cp_{3}-{\hat{V}}_{0}{\hat{T}}_{2}cp_{0}-Q{)}^{2} \end{array}$$

(37) $$s.t.{\hat{\alpha }}_{\min}\leq \hat{\alpha }\leq {\hat{\alpha }}_{\max}$$

where $Y_{2}$ is the objective of the optimization problem; $\hat{\alpha }$ represents the estimated value of percentage for ZnS after reconciliation; ${\hat{\alpha }}_{\min}$ and ${\hat{\alpha }}_{\max}$ denote the bottom and top bounds of $\hat{\alpha }$ respectively. Through the iterative process of the above steps, greater accuracy and a smaller range of ZnS fluctuation in the feed can be obtained. Therefore, staff can better understand changes of the feeding composition and make adjustments as required.

## 5. Results from Real Industrial Data

**Figure 9:**
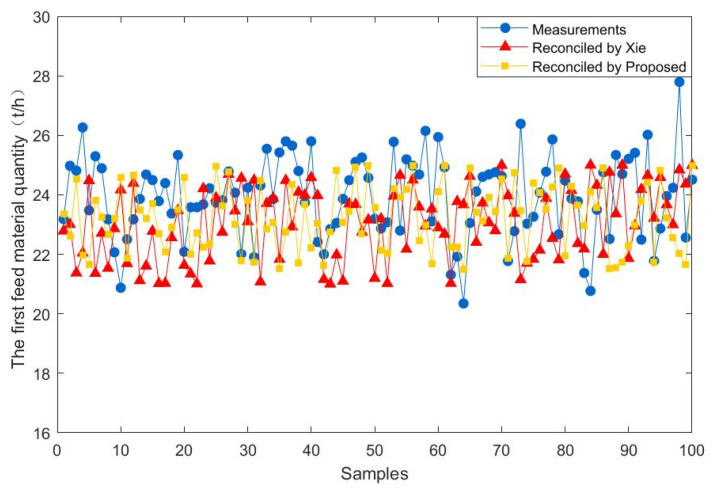
Reconciled results for the first feed material quantity.

**Figure 10:**
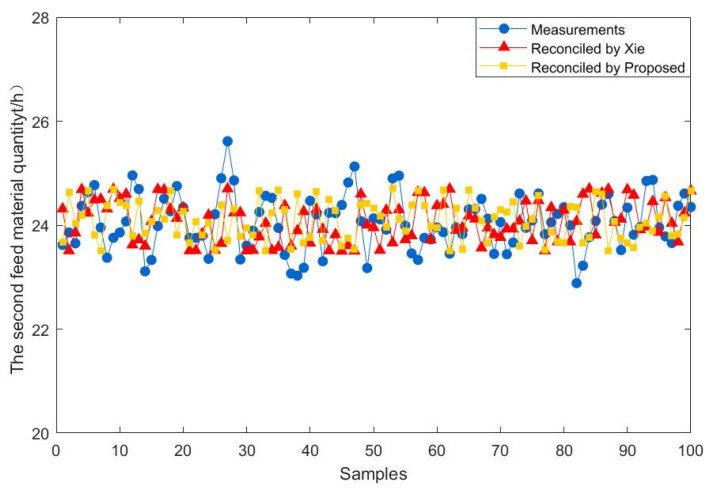
Reconciled results for the second feed material quantity.

**Figure 11:**
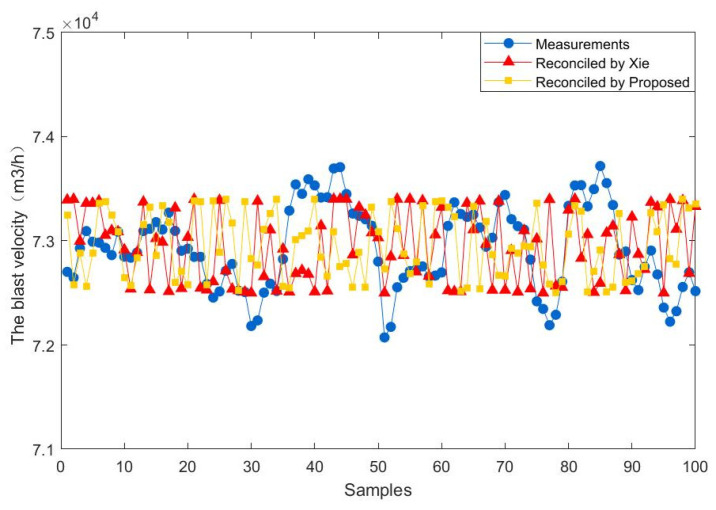
Reconciled results for the blast velocity.

**Figure 12:**
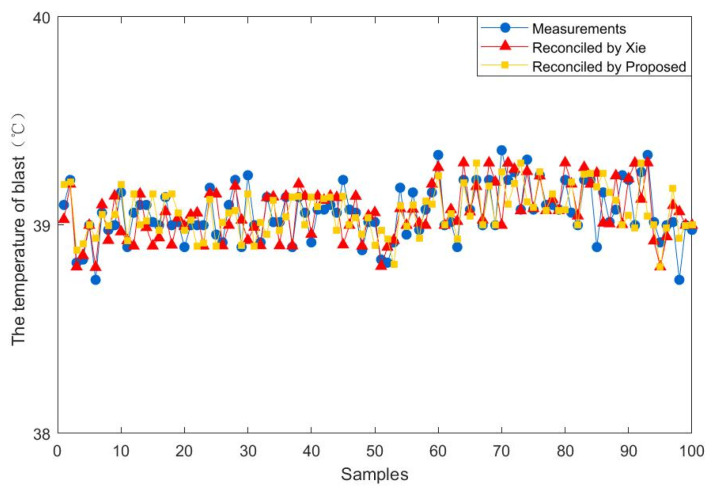
Reconciled results for the temperature of blast.

**Figure 13:**
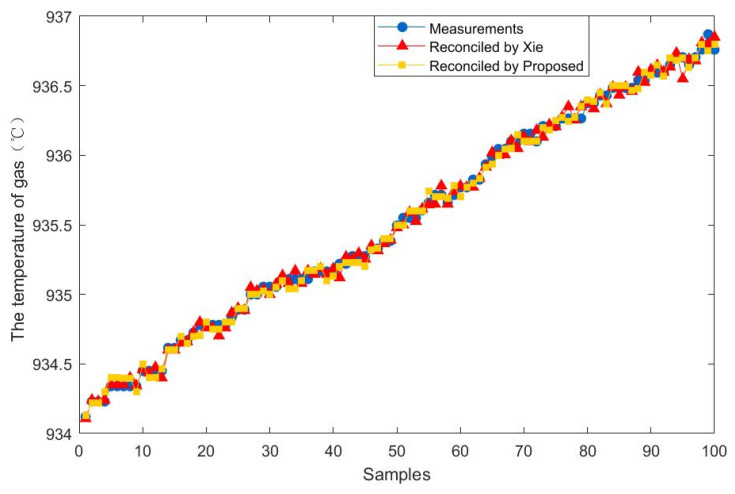
Reconciled results for the temperature of gas.

**Figure 14:**
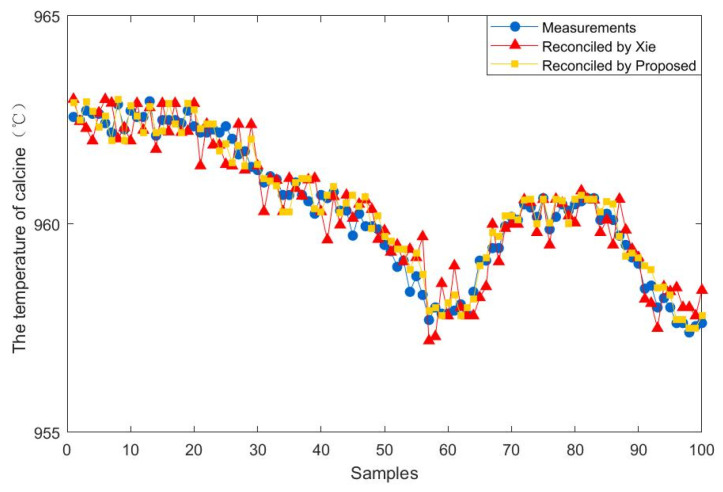
Reconciled results for the temperature of calcine.

To further demonstrate the effectiveness of the proposed method, 100 steady-state samples are collected from the real industry of a fluidized bed roaster. In addition, the data reconciliation results of the proposed estimator are compared with those of Xie estimator. The results of the comparison between the two methods are presented in Figure 9, Figure 10, Figure 11, Figure 12, Figure 13 and Figure 14, which respectively present the reconciled results for the first feeding quantity, the second feeding quantity, blast velocity, blast temperature, gas temperature and calcine temperature. From Figure 9, Figure 10, Figure 11, Figure 12, Figure 13 and Figure 14, it is known that the reconciled results by the novel robust estimator have a smaller range of fluctuation than that obtained by Xie estimator. It shows that whatever the uncertainty of the fluctuation of the measurements and the number of gross errors they contain, the data reconciliation approach based on the novel robust estimator is highly robust.

**Figure 15:**
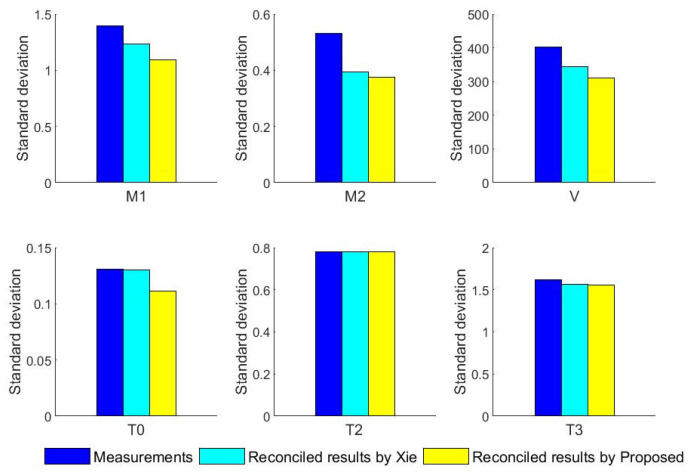
The standard deviation of reconciled results and measurements.

In addition, the standard deviations of the reconciled results and measurements for six variables obtained from the two methods are calculated, and the results of the comparison are presented in Figure 15. Figure 15 shows that, for the first feeding quantity, the seconding feed quantity, the blast velocity and the temperature of blast, the standard deviation of the reconciled results based on the novel robust estimator is less than Xie. For the temperature of gas and calcine, the standard deviations of reconciled results for the two methods are closer, because the measurement fluctuation ranges for both two variables are all very small. The results demonstrate that the data reconciliation based on the novel robust estimator provides better reconciliation results for variables with a large fluctuation range.

**Figure 16:**
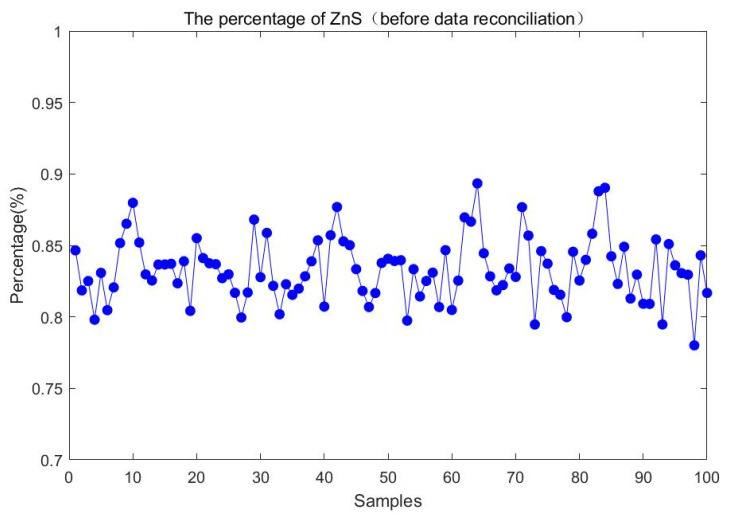
The percentage of ZnS before data reconciliation.

**Figure 17:**
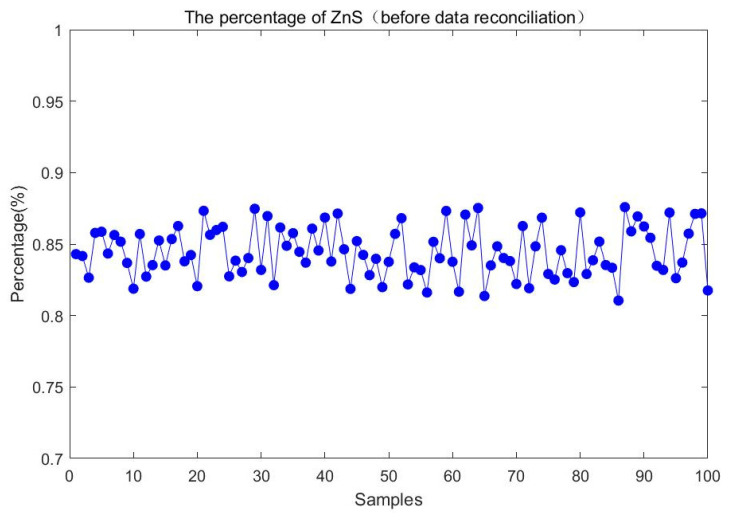
The percentage of ZnS after data reconciliation.

For the change in feeding composition, the above 100 steady-state samples are also collected to compare the percentage of ZnS before and after data reconciliation. Experimental results are shown in Figure 16 and Figure 17. It is evident that the ZnS fluctuation range obtained by the proposed method is smaller than that before data reconciliation. It illustrates that the percentage of ZnS estimated by the reconciled results is more accurate and could better reflect the change of feeding composition over a period of time.

## 6. Conclusions

The feeding composition in an industrial process can better reflect the state of production. In this paper, estimation of feeding composition based on robust data reconciliation is proposed to account for the unknown change in feeding composition. According to the robust estimation theory, a novel robust estimator is presented to handle the measurements with random errors and gross errors. In comparison with the objective function and influence function of other robust estimators, the proposed estimator is proved to be more robust. Besides, the linear and nonlinear numerical examples are used to further verify the effectiveness of the estimator. Then, an iterative robust hierarchical data reconciliation and estimation of feeding composition strategy is proposed and used for a fluidized bed roaster. The reconciled and estimated results show that the proposed strategy is beneficial for the estimation of feeding composition in the actual industrial process.

## Figures and Tables

**Table 1:** Tuning parameters of three estimators at distinct relative efficiency levels.

	M-Estimator	Tuning Parameter
1	Welsch	$\left\{ \begin{array}{l} c_{w}=2.3828\;\;\;\;\;E_{ff}=90\%\\ c_{w}=2.9846\;\;\;\;\;E_{ff}=95\%\\ c_{w}=3.9077\;\;\;\;\;E_{ff}=98\%\\ c_{w}=4.7343\;\;\;\;\;E_{ff}=99\% \end{array}\right.$
2	Xie	$\left\{ \begin{array}{l} c_{x}=1.6705\;\;\;\;\;E_{ff}=90\%\\ c_{x}=1.9597\;\;\;\;\;E_{ff}=95\%\\ c_{x}=2.3409\;\;\;\;\;E_{ff}=98\%\\ c_{x}=2.6359\;\;\;\;\;E_{ff}=99\% \end{array}\right.$
3	Proposed	$\left\{ \begin{array}{l} c_{p}=1.3082\;\;\;\;\;E_{ff}=90\%\\ c_{p}=1.5424\;\;\;\;\;E_{ff}=95\%\\ c_{p}=2.0942\;\;\;\;\;E_{ff}=98\%\\ c_{p}=2.6060\;\;\;\;\;E_{ff}=99\% \end{array}\right.$

**Table 2:** Reconciled results of different methods with two gross errors.

Stream	True	Original Meas.	Meas. with Gross Error	Proposed	Xie	Welsch	Cauchy	Fair
$x_{1}$	5	4.995	4.995	5.0111	5.0152	5.0346	5.0519	5.1777
$x_{2}$	15	14.91	16.91	15.0420	15.0540	15.1202	15.1970	15.6413
$x_{3}$	15	15.01	15.01	15.0420	15.0540	15.1202	15.1970	15.6413
$x_{4}$	5	5.002	5.002	4.9987	4.9985	5.0053	5.0230	5.1795
$x_{5}$	10	9.98	10.98	10.0433	10.0555	10.1149	10.1740	10.4618
$x_{6}$	5	5.019	5.019	5.0322	5.0403	5.0803	5.1221	5.2841
$x_{7}$	5	5.014	5.014	5.0111	5.0152	5.0346	5.0519	5.1777
SSE	--	--	--	0.0067	0.0110	0.0510	0.1287	1.2117
TER	--	--	--	0.9424	0.9263	0.8457	0.7606	0.2953
RER	--	--	--	0.9101	0.8838	0.7501	0.6008	−0.2626

**Table 3:** Reconciled results of different methods with three gross errors.

Stream	True	Original Meas.	Meas. with Gross Error	Proposed	Xie	Welsch	Cauchy	Fair
$x_{1}$	5	4.995	4.995	5.0080	5.0164	5.0574	5.0323	4.3533
$x_{2}$	15	14.91	16.91	15.0390	15.0552	15.1425	15.1802	15.1833
$x_{3}$	15	15.01	15.01	15.0390	15.0552	15.1425	15.1802	15.1833
$x_{4}$	5	5.002	5.002	4.9991	4.9984	5.0037	5.0244	5.3066
$x_{5}$	10	9.98	10.98	10.0399	10.0568	10.1388	10.1558	9.8767
$x_{6}$	5	5.019	5.019	5.0320	5.0404	5.0814	5.1235	5.5233
$x_{7}$	5	5.014	3.514	5.0080	5.0164	5.0574	5.0323	4.3533
SSE	--	--	--	0.0058	0.0115	0.0731	0.1071	1.2866
TER	--	--	--	0.9743	0.9642	0.9111	0.8954	0.3474
RER	--	--	--	0.9641	0.9470	0.8621	0.8446	0.1268

**Table 4:** Reconciled results of different methods for nonlinear constraints.

Stream	Standard Deviation	True	Meas.	Proposed	Xie	Welsch	Cauchy	Fair
$x_{1}$	0.5	4.5124	4.5360	4.5378	4.5280	4.5379	4.5796	4.4727
$x_{2}$	0.6	5.5819	5.9070	5.5754	5.5655	5.5331	5.5360	5.6514
$x_{3}$	0.2	1.9260	1.8074	1.9221	1.9223	1.9200	1.9153	1.9321
$x_{4}$	0.2	1.4560	1.4653	1.4653	1.4842	1.4924	1.5096	1.4655
$x_{5}$	0.5	4.8545	4.5491	4.8156	4.8010	4.8083	4.7882	4.7870
$u_{1}$	--	11.070	--	11.0988	11.1178	11.1939	11.2079	10.9076
$u_{2}$	--	0.6147	--	0.6143	0.6160	0.6187	0.6168	0.6104
$u_{3}$	--	2.0504	--	2.0469	2.0349	2.0317	2.0345	2.0372
SSE	--	--	--	0.0031	0.0067	0.0222	0.0333	0.0377
TER	--	--	--	0.8950	0.8192	0.7751	0.6631	0.7994
RER	--	--	--	0.8805	0.8008	0.7324	0.5930	0.7693

## Data Availability

Publicly available datasets were analyzed in this study. It can be found here: https://open.library.ubc.ca/cIRcle/collections/ubctheses/831/items/1.0078522 (accessed on 1 February 2021).
